# Cytomegalovirus induced refractory TTP in an immunocompetent individual: a case report

**DOI:** 10.1186/s12879-019-4037-9

**Published:** 2019-05-08

**Authors:** Medhini Boteju, Praveen Weeratunga, Ahalyaa Sivashangar, Thashi Chang

**Affiliations:** 1 University Medical Unit, National Hospital of Sri Lanka, Colombo, Sri Lanka; 2 Department of Clinical Medicine, Faculty of Medicine, University of Colombo, 25, Kynsey Road, Colombo, 00800 Sri Lanka

**Keywords:** Cytomegalo-virus, Refractory thrombotic thrombocytopenic purpura

## Abstract

**Background:**

Thrombotic thrombocytopenic purpura (TTP) is a rare, potentially fatal disease with multisystem involvement. Cytomegalovirus (CMV) infection as a cause of refractory TTP, has been reported only in immunocompromised individuals. We report a case of CMV-induced refractory TTP in an immunocompetent individual.

**Case presentation:**

A 35-year-old, previously healthy Sri Lankan man, presented with fever for 3 days with gum bleeding and progressive drowsiness. His Glasgow coma scale score was 10/15. He did not have papilloedema or neck stiffness. Laboratory evaluation showed a severe thrombocytopenia with microangiopathic haemolytic anaemia. There was marginal renal impairment and normal coagulation profile. Non-contrast CT scan of brain was normal. A diagnosis of thrombotic thrombocytopenic purpura was made. Despite daily plasma exchanges and high-dose steroids, he failed to achieve the expected therapeutic response, thus demonstrating refractory TTP. On exploring for possible causes of refractoriness to treatment, a clinically significant PCR titre of CMV was detected. Treatment of CMV infection lead to complete recovery of TTP. His disease course was further complicated with spontaneous spinal haemorrhage leading to neurological sequelae.

**Discussion and conclusions:**

This is the first report of CMV induced refractory TTP in an immunocompetent adult. It is also the first report of clinically significant spontaneous spinal haematoma in TTP. These two rare occurrences should be considered when patients with refractory TTP do not improve as expected.

## Background

Thrombotic thrombocytopenic purpura (TTP) first described by Dr. E Moschcowitz in 1924, is a rare, potentially fatal disease with multisystem involvement. TTP is characterized by a pentad of features including thrombocytopenia, microangiopathic haemolytic anaemia, neurological abnormalities, renal failure and fever [1]. Plasma exchange and immunosuppression have been found to be effective treatment in patients with TTP. In a retrospective analysis of 11-year experience on TTP, 93% were reported to have excellent response to early initiation of plasma exchange while 86% reached complete remission [2]. Similarly, 55% of patients achieved remission with corticosteroids alone, in a study conducted prior to systematic use of plasma exchange in TTP [3]. However, a proportion of patients with TTP remain refractory to treatment and are often found to have an underlying secondary aetiology that drives the TTP. Reported secondary causes of refractory TTP include CMV and other viral infections, ventilator-associated or urinary tract-associated gram-negative sepsis, central line-associated staphylococcal bacteraemia, and occult malignancies [4]. However, CMV induced refractory TTP has been previously reported only in immunocompromised individuals. Herein we report a case of CMV induced refractory TTP in an immunocompetent individual.

## Case presentation

A previously healthy 35-year-old Sri Lankan man presented with high-grade intermittent fever for 3 days with constitutional symptoms. He had spontaneous gum bleeding with no other overt bleeding manifestations. He had associated intermittent frontal headache of moderate severity at presentation and subsequently developed gradually worsening drowsiness. Rest of the history including his past medical and family histories were unremarkable. In particular, he did not have diabetes mellitus, history of recurrent infections, unprotected sexual contact or recreational drug abuse.

On examination, his body temperature was 100 °F and his Glasgow coma scale score was 10/15. He was mildly pale. There was no neck stiffness. Fundoscopic examination was normal. There were no focal neurological signs. His pulse rate was 100 bpm and blood pressure was 130/80 mmHg, Rest of the cardiovascular, respiratory and abdominal examinations were normal.

His full blood count showed a white cell count of 9.2 × 10^3^/dl (Normal Range (NR) 4–11 × 10^3^) with neutrophil predominance (77%). Haemoglobin was 6.9 g/dl (NR 11–15) and platelet count on admission was 7 × 10^3^ (NR150–400 × 10^3^). His coagulation profile was normal with prothrombin time of 12.8 s (NR 10–13) and APTT 30 s (NR 26–40). Serum creatinine was slightly elevated at 137 mmol/l (NR 60–120 umol/L) and the electrolytes were normal. There was indirect hyperbilirubinaemia with total bilirubin of 44.7 μmol/l (NR 1.7–20.5) and direct bilirubin of 7.3 μmol/l (NR 1.7–5.1). The serum lactate dehydrogenase level was 3115 U/l (NR 160–450). Direct coombs test and dengue serology were negative. Non-contrast CT scan of the brain was normal. Blood picture showed evidence of severe thrombocytopenia with microangiopathic haemolytic anaemia (MAHA).

A diagnosis of TTP was made and he was promptly commenced on therapeutic plasma exchange and 1 mg/kg of oral prednisolone. However, he developed two episodes of generalized tonic-clonic convulsions which progressed in to a non-convulsive status epilepticus on the fifth day of illness, which required elective ventilation in the intensive care unit. On day 18, he developed flaccid paraparesis with sphincter dysfunction. Magnetic resonance imaging showed haemorrhage at multiple spinal levels including cervical spine sub-arachnoid space, anterior epidural space and intra-thecal space from T10 to L3 vertebral levels, and in the the region of the conus medullaris.

Surgical drainage of the spinal haematoma was considered hazardous. At this point his platelet count was 85 × 10^3^ and remained < 100 × 10^3^ with  MAHA persisting despite 16 plasma exchanges and high dose steroids.

Initially, the refractoriness of the TTP was attributed to ventilator associated pneumonia. However, since successful treatment of sepsis did not improve the MAHA, an alternative aetiology was investigated. Polymerase chain reaction of serum revealed 2100 IU/ml copies of CMV (Reference laboratory cutoff value more than 640 IU/ml was considered clinically significant). CMV DNA was quantitatively determined by RealStar® CMV PCR Kit 1.0 (Altona diagnostics). Screening tests for autoimmune diseases, other chronic infections and immunodeficiency, which included ANA, serum complement levels, serum immunoglobulin levels, HIV 1, HIV 2, VDRL, hepatitis screen and HbA1c were negative.

The patient was commenced on oral valganciclovir 450 mg/daily and continued for 21 days. After about 6 days of valganciclovir treatment his platelet count increased to 198 × 10^3^/cumm and the MAHA resolved. After resolution of the TTP, the patient was transferred to a rehabilitation facility for further care. At three months’ review, he had normal haematological and biochemical parameters and a negative PCR quantification of CMV. He had regained ability to walk with support and had normal sphincter function.

## Discussion

This is the first report of CMV induced refractory TTP in an immunocompetent individual. All previous reports of CMV induced TTP had been in immunocompromised hosts [5–7]. Our case report also highlights the occurrence of spinal neurological deficits as a complication of haemorrhage related to TTP.

Refractory TTP in our patient was diagnosed when immunosuppression and plasma exchange failed to produce a doubling of platelet count after 4 days [8]. When a patient fails to respond to standard treatment, it is essential to look for an underlying secondary aetiology that drives the TTP. The usual causes include systemic infection, malignancy and treatment related complications [9]. When our patient did not have the expected rise in platelet count despite successful treatment of sepsis, further investigations for occult infections, autoimmune diseases and malignancies led to the discovery of a significant titre of CMV in the blood. Resolution of TTP with treatment of CMV proved that it was indeed the cause of the refractory TTP. However, extensive evaluation did not reveal evidence of any immunodeficiency.

Symptomatic CMV infection in immunocompetent adults is rare. Malaise, fever, lymphadenopathy, glandular fever–like illness were the commonly reported clinical features in immunocompetent patients with CMV [10, 11]. Most have presented with acute hepatitis [12, 13]. Rarely some have presented with neurological and intestinal manifestations including encephalitis, transverse myelitis, oesophagitis, colitis and multiple gastro-intestinal tract ulcers [14–16]. In contrast, CMV infection was commonly reported among immunocompromised patients including transplant recipients and those with acquired immunodeficiency syndrome. Patients deficient in cell-mediated immunity are at higher risk as the CMV specific CD4+ and CD8+ cell functions are impaired. These cells play a pivotal role in protection after primary infection and preventing reactivation of latent disease [17]. TTP was among recognized clinical manifestations of CMV infection in immunocompromised adults. [5–7]. The postulated mechanisms for CMV induced TTP include direct and cytokine-mediated endothelial damage which result in increased expression of endothelial adhesion molecules and release of Von-Willebrand factor [18].

The course of illness in our patient was further complicated with spontaneous spinal epidural and intrathecal haematoma, which resulted in flaccid paraparesis. Neurological complications in TTP are most commonly explained by micro-thrombi resulting brief episodes of focal ischaemia [19]. The reported MRI findings in TTP include cerebral cortical infarcts and haemorrhagic infarcts [20, 21]. Clinically important central nervous system bleeding has been reported in TTP [22], but is exceptionally rare despite low platelet counts and has grave prognosis. Ours is the first report of clinically significant spontaneous spinal haematoma in TTP.

## Conclusions

CMV infection is a rare but treatable cause of refractory TTP, most commonly occurring in immunocompromised individuals, but as highlighted in our report, also occurring rarely in immunocompetent adults. Awareness and early identification of CMV is essential in the successful treatment of refractory TTP and to avoid unnecessary prolongation of illness with inherent hazard to life. This case report also highlights the need for vigilance to detect haemorrhage as a potential cause for neurological sequelae in addition to thrombotic microangiopathic complications in TTP.

## Abbreviations

ANA: Anti-nuclear antibody
APTT: Activated partial thromboplastin time
CD: Cluster of differentiation
CMV: Cytomegalovirus
HIV: Human immunodeficiency virus
ICU: Intensive care unit
MAHA: Microangiopathic haemolytic anaemia
MRI: Magnetic resonance imaging
PCR: Polymerase chain reaction
TTP: Thrombotic thrombocytopenic purpura
VDRL: Venereal Disease Research Laboratory
